# Tenecteplase in large-vessel occlusion with or without thrombectomy: a meta-analysis

**DOI:** 10.3389/fneur.2025.1715049

**Published:** 2026-01-13

**Authors:** Ahmed Alkhiri, Aser F. Alamri, Fahad Alturki, Remas M. Alonazi, Thanh N. Nguyen, Fahad S. Al-Ajlan, Adel Alhazzani

**Affiliations:** 1 Neuroscience Center of Excellence, King Faisal Specialist Hospital and Research Centre, Riyadh, Saudi Arabia; 2 College of Medicine, Imam Mohammad Ibn Saud Islamic University, Riyadh, Saudi Arabia; 3 Department of Radiology, School of Medicine, Boston University, Boston, MA, United States; 4 Department of Neurology, School of Medicine, Boston University, Boston, MA, United States; 5 Alfaisal University, Riyadh, Saudi Arabia

**Keywords:** bridging, large-vessel occlusion, stroke, tenecteplase, thrombectomy

## Abstract

**Background:**

Despite the growing adoption of Tenecteplase for stroke thrombolysis, key questions such as the role of bridging therapy and extended-window use in large-vessel occlusion (LVO) patients with or without access to endovascular thrombectomy (EVT) remain unresolved.

**Methods:**

We conducted a systematic review of randomized clinical trial (RCT) data involving LVO patients who were treated with or without Tenecteplase. Outcomes were stratified by EVT status, and further subgroup analysis based on treatment window (≤4.5 vs. > 4.5 h) was conducted. Generalized odds ratios (gORs) were used to assess disability reduction by modified Rankin Scale (mRS) shift analysis. Risk ratios (RRs) with 95% confidence intervals (CIs) were calculated for functional independence (mRS 0–2), excellent outcome (mRS 0–1), mortality, and symptomatic intracranial hemorrhage (sICH).

**Results:**

Five studies (2,054 patients) were included. Treatment with Tenecteplase was associated with greater disability reduction (gOR, 1.19; 95% CI, 1.06–1.34), more excellent outcome (RR, 1.21; 95% CI, 1.06–1.38) and functional independence (RR, 1.14; 95% CI, 1.03–1.26) compared to no thrombolysis. Rates of sICH (RR, 1.46; 95% CI, 0.87–2.54) and mortality (RR, 1.07; 95% CI, 0.84–1.36) were similar between groups. Before EVT, Tenecteplase within 4.5 h was associated with greater disability reduction and higher functional independence compared with EVT alone; however, no benefit was observed beyond 4.5 h in those undergoing EVT. Among LVO patients not treated with EVT but with salvageable tissue on CT perfusion, Tenecteplase administered after 4.5 h was associated with improved functional outcomes.

**Conclusion:**

In this meta-analysis, Intravenous Tenecteplase was associated with improved outcomes when administered before EVT within 4.5 h and in the extended window, guided by advanced neuroimaging, among selected patients who lacked access to EVT.

**Systematic review registration:**

CRD420251115406.

## 1 Introduction

Intravenous thrombolysis (IVT) and endovascular thrombectomy (EVT) are established treatments for acute ischemic stroke, providing time-dependent benefits (1, 2). Over the past two decades, Tenecteplase has emerged as a practical alternative or replacement to alteplase. Multiple trials have demonstrated the non-inferiority of Tenecteplase 0.25 mg/kg compared to Alteplase 0.9 mg/kg, with a meta-analysis suggesting more excellent outcomes (modified Rankin Scale [mRS] 0–1) with Tenecteplase (3). Consequently, the global adoption of Tenecteplase for stroke thrombolysis has been steadily increasing. On March 4, 2025, the U. S. FDA approved Tenecteplase 0.25 mg/kg for this indication, based on the design and outcomes of the Canadian AcT (Alteplase Compared to Tenecteplase) trial (4).

Several guidelines currently recommend IVT before EVT for eligible patients with LVO (5, 6). In a pooled analysis of six trials from the Improving Reperfusion Strategies in Acute Ischemic Stroke (IRIS) collaboration comparing IVT plus EVT with EVT alone, neither the non-inferiority of EVT alone nor the superiority of combined therapy for functional independence was demonstrated. However, a time-dependent benefit of bridging IVT was observed, with significant advantages when treatment was administered within 140 min of symptom onset. In the IRIS cohort, 97.8% of patients in the bridging arm were treated with Alteplase (7).

Tenecteplase for patients with LVO has been studied in different settings, including trials comparing it with alteplase or with no thrombolysis. Patients with LVO were also enrolled as a part of unselected stroke patients in large non-inferiority trials of Tenecteplase versus Alteplase (4, 8). The EXTEND-IA TNK part I (Tenecteplase versus Alteplase before Endovascular Therapy for Ischemic Stroke) trial showed that before EVT and within 4.5 h, Tenecteplase increased both reperfusion and favorable functional outcome rates compared to Alteplase (9). In the AcT trial, 520 of 1,577 patients (33.0%) had LVO, and in this subgroup, functional, reperfusion, and safety outcomes were similar between Tenecteplase and alteplase (10). The BRIDGE TNK (Thrombectomy with versus without TNK in Stroke) trial showed that Tenecteplase administered before EVT within 4.5 h significantly improved functional independence compared to EVT alone (11).

Beyond 4.5 h, the TRACE III (Tenecteplase Reperfusion Therapy in Acute Ischemic Cerebrovascular Events–III) trial showed that among patients with LVO who lacked access to EVT, Tenecteplase was associated with a higher likelihood of excellent functional outcomes compared with no thrombolysis (12). More recently, the ETERNAL-LVO (Efficacy of Tenecteplase in Large Vessel Occlusion Stroke Within 24 Hours of Symptom Onset) trial (13) did not demonstrate a benefit of Tenecteplase compared to medical management (82% received alteplase) in patients with LVO treated within 24 h of symptom onset.

Given the heterogeneity in the available literature and the growing use of Tenecteplase as a primary thrombolytic agent, we conducted a systematic review and meta-analysis to evaluate its benefit–risk profile compared with no thrombolysis in patients with LVO (with and without EVT and within and beyond 4.5 h) and to provide important insights into the role of Tenecteplase in this population.

## 2 Methods

This meta-analysis included only previously published studies and was exempt from institutional review board approval. All data analyzed are contained within this article. The protocol was registered with PROSPERO (CRD420251115406) and followed the Preferred Reporting Items for Systematic Reviews and Meta-Analyses (PRISMA) reporting guideline (14).

### 2.1 Information source and search strategy

We searched MEDLINE/PubMed, Embase, Cochrane Library, and Web of Science using combinations of terms related to stroke, LVO, Tenecteplase, and EVT. The final searches were conducted in November 2025. Full search strategies are provided in the Supplementary material. To ensure comprehensive coverage, we also conducted manual searches, including screening reference lists, forward citation tracking, reviewing prior narrative and systematic reviews on Tenecteplase for acute ischemic stroke, and consulting senior stroke experts.

### 2.2 Study selection and eligibility

After duplicate removal, two independent reviewers (AhA and AFA) screened titles and abstracts to identify potentially eligible studies. Full texts of selected reports were subsequently assessed using prespecified inclusion criteria. We included randomized clinical trials (RCTs) and secondary analyses of RCTs that evaluated efficacy or safety outcomes of patients with LVO treated with intravenous Tenecteplase (0.25 mg/kg) versus no thrombolysis. To ensure clinical relevance to the emerging use of Tenecteplase as a primary thrombolytic agent, we restricted inclusion to studies in which all or the majority of participants in the comparator group did not receive alternative thrombolytic drugs. Studies were required to specify whether EVT was performed and to report outcomes stratified by EVT status. No restrictions were applied to the treatment time window. Observational studies, conference abstracts, and non-English publications were excluded.

### 2.3 Data extraction and outcomes

Two authors (AhA and FA) independently extracted study and patient characteristics using standardized forms. Data on efficacy and safety outcomes were collected. Efficacy outcomes included disability reduction (shift in the mRS score), rates of excellent outcome (mRS 0–1 at 90 days), favorable outcome (mRS 0–2), and good outcome (mRS 0–3). Safety outcomes included symptomatic intracerebral hemorrhage (sICH) and 90-day mortality.

### 2.4 Risk of bias

Risk of bias for randomized trials was assessed using the Cochrane Risk of Bias 2 (RoB 2) tool, which evaluates five domains and classifies overall risk as low, some concerns, or high (15). For the trial emulation analysis, we used the ROBINS-I (Risk of Bias in Non-Randomized Studies of Interventions) tool, which assesses bias across seven domains: confounding, selection of participants, classification of interventions, deviations from intended interventions, missing data, measurement of outcomes, and selection of the reported result. Each domain is rated as low, moderate, serious, or critical risk of bias, contributing to an overall judgment for each study (16). Two reviewers (FA and RMA) independently conducted all assessments, with discrepancies resolved by consensus or consultation with the senior author.

### 2.5 Statistical analysis

Statistical analyses were conducted using RevMan version 5.4. For the study by Altersberger et al. (17), only patients treated with Tenecteplase at a dose of 0.25 mg/kg were included, while those receiving 0.40 mg/kg were excluded to enhance the homogeneity of the pooled analysis. Disability was assessed using mRS shift analysis, with generalized odds ratios (gORs) calculated via the generic inverse variance method. Risk ratios (RRs) were calculated for binary outcomes, including functional independence (mRS 0–2), excellent outcome (mRS 0–1), sICH, and 90-day mortality. Effect estimates with 95% CIs were generated using a fixed-effect model because few studies contributed to each analysis (18, 19). When available, adjusted effect measures were preferentially used. Heterogeneity was evaluated using the *Q* statistic and *I*^2^, with *p* < 0.05 or *I*^2^ > 50% considered indicative of significant heterogeneity. Assessment of publication bias was not feasible as fewer than 10 articles were included in each analysis (20).

Sensitivity analyses were performed for the primary analysis by excluding the CHABLIS-T II trial, as a small proportion in the comparator arm received thrombolytic therapy (23.1% IV alteplase and 1.8% urokinase) (21). Prespecified subgroup analyses were conducted according to endovascular thrombectomy status and time window.

## 3 Results

Of 695 records screened, five studies (2,054 patients) were included (11, 12, 17, 21, 22) with 1,000 receiving Tenecteplase. The study flowchart and study selection criteria are shown in Supplementary Figure S1. Table 1 summarizes study characteristics. The BRIDGE TNK trial (11) and a target trial emulation analysis based on the SWIFT DIRECT and EXTEND-IA TNK Parts 1 and 2 trials (17) included patients treated within 4.5 h. In the target trial emulation study, two doses of Tenecteplase (0.4 mg/kg and 0.25 mg/kg) were evaluated. Only patients who received Tenecteplase at a dose of 0.25 mg/kg were included in this study (17). The TIMELESS, CHABLIS-T II, and TRACE III trials (12, 21, 22) included patients treated with Tenecteplase after 4.5 h. All included studies included patients with anterior circulation occlusion, except for the BRIDGE TNK trial (11), which included 49 patients (9%) with vertebrobasilar occlusion.

**Table 1:** Characteristics of included studies.

Author	Design	Time window	Imaging selection	Included vessels	Total patients	Total TNK	Treated with EVT		
							Total	TNK	EVT alone
Qiu et al. (2025) (11)	BRIDGE-TNK trial	4.5 h	Standard imaging	ICA, M1, M2 and BA	550	278	550 (100%)	278	272
Xiong et al. (2024) (12)	TRACE III trial	4.5–24 h	CTP	ICA, M1 and M2	516	264	0 (0%)	-	-
Albers er al. (2024) (22)	TIMELESS trial	4.5–24 h	CTP	ICA, M1 and M2	458	228	351 (76.6%)	174	177
Cheng et al. (2025) (21)	CHABLIS-T II	4.5–24 h	CTP	ICA, M1, M2 and ACA	224	111	122 (54.5%)	58	64
Altersberger et al. (2025) (17)	Target trial emulation analysis based on SWIFT DIRECT and EXTEND-IA TNK Parts 1 and 2 trials	4.5 h	Standard imaging	ICA and M1	306	119	306 (100%)	119	187

TNK, tenecteplase; EVT, endovascular thrombectomy; CTP, computed tomography perfusion; ICA, internal carotid artery; M1, first segment of the middle cerebral artery; M2, second segment of the middle cerebral artery; BA, basilar artery; ACA, anterior cerebral artery.

Risk of bias assessments are detailed in Supplementary Table S1 for RCTs and Supplementary Table S2 for the trial emulation analysis. Briefly, CHABLIS-T II and BRIDGE-TNK were assessed as having some concerns, whereas TIMELESS and TRACE III were judged to be at low risk of bias. The trial emulation analysis was determined to be at moderate risk of bias.

**Figure 1:**
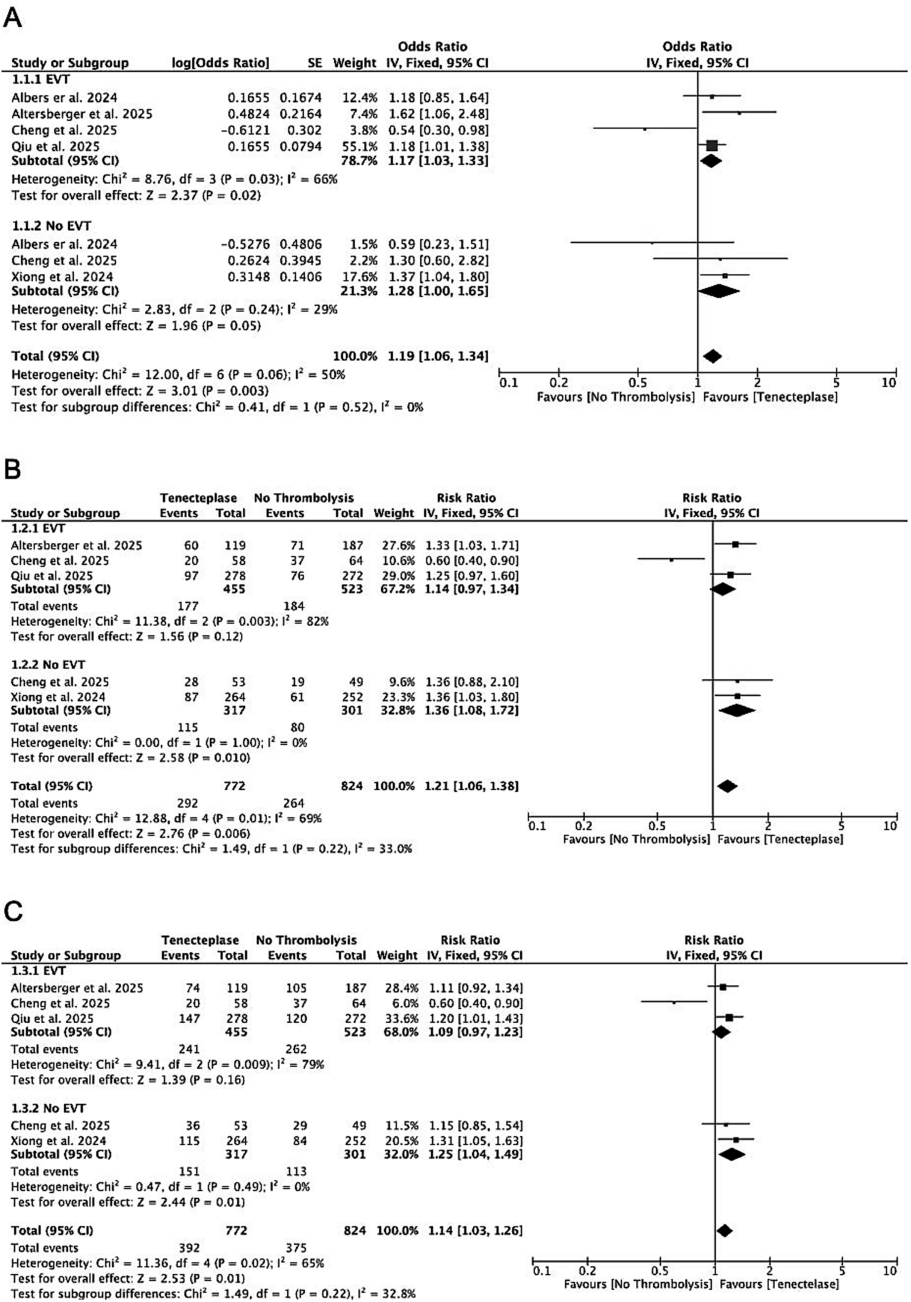
Forest plots of overall treatment effects comparing intravenous tenecteplase with no thrombolysis among patients with LVO, showing mRS shift analysis **(A)**, excellent functional outcome (mRS 0–1) **(B)**, and functional independence (mRS 0–2) **(C)**.

For patients with LVO, there was greater disability reduction (mRS shift: gOR 1.19, CI 1.06–1.34; *p* = 0.003; *I*^2^ = 50%) with Tenecteplase compared to no thrombolysis. Similarly, patients receiving Tenecteplase had higher excellent outcomes (mRS 0–1: RR 1.21, CI 1.06–1.38; *p* = 0.006; *I*^2^ = 69%) and functional independence (mRS 0–2: RR 1.14, CI 1.03–1.26; *p* = 0.01; *I*^2^ = 65%) compared to those treated without thrombolysis (Figure 1). Rates of sICH (RR 1.47, CI 0.87–2.49; *p* = 0.15; *I*^2^ = 39%) and mortality (RR 1.07, CI 0.84–1.36; *p* = 0.60; *I*^2^ = 0%) did not differ significantly between groups (Supplementary Figure S2). In each analysis, no significant interaction was observed between patients who underwent subsequent EVT and those who did not (test of subgroup differences, *p* > 0.05; *I*^2^ < 50%). Sensitivity analyses showed similar results after exclusion of the CHABLIS-T II trial (Supplementary material).

**Figure 2:**
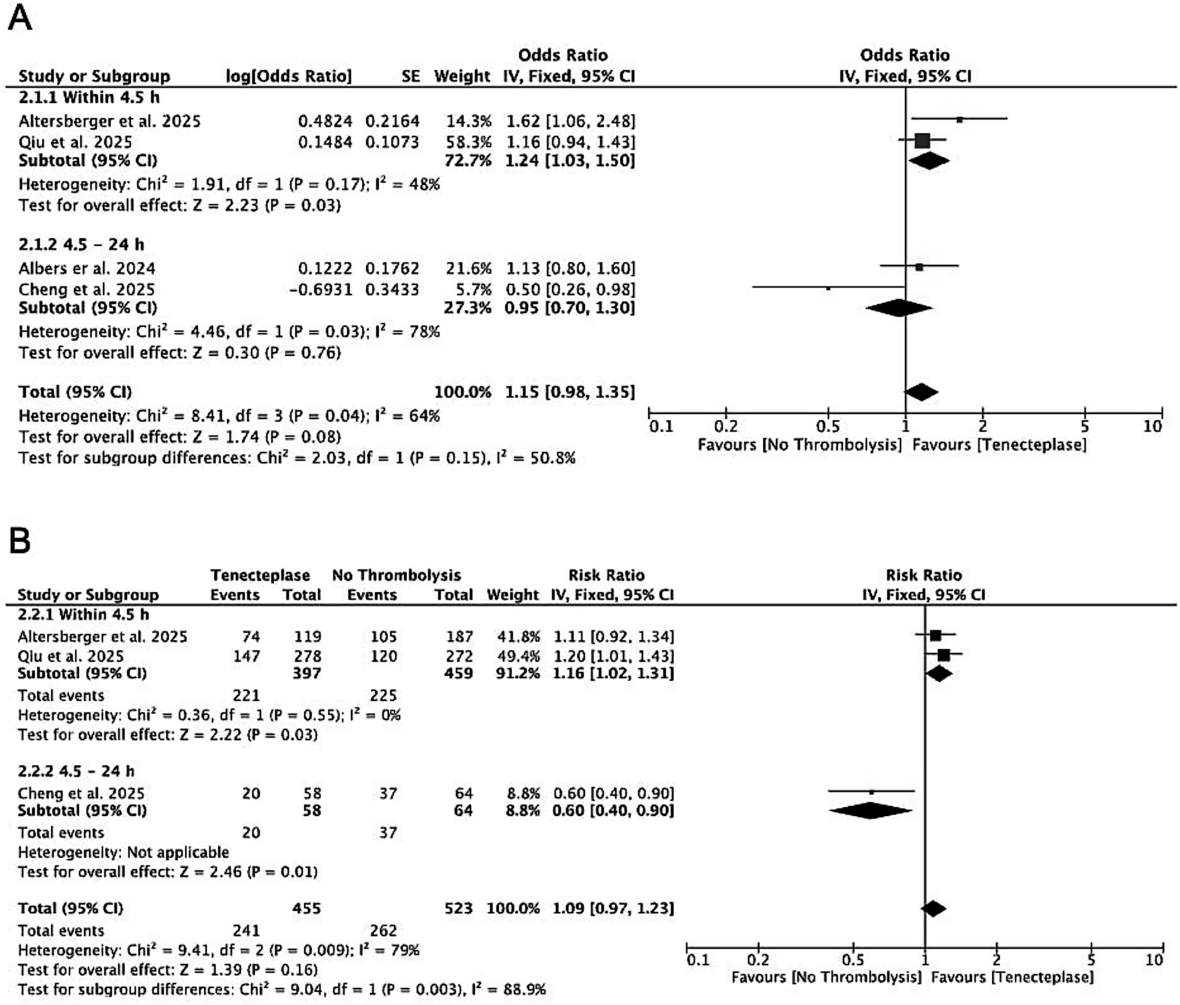
Forest plots of subgroup analyses by treatment timing among patients with LVO undergoing EVT, showing mRS shift analysis **(A)** and functional independence (mRS 0–2) **(B)**.

Figure 2 and Supplementary Figure S3 present subgroup outcomes stratified by treatment timing and EVT status. For patients who underwent EVT within 4.5 h, Tenecteplase plus EVT was associated with greater disability reduction (mRS shift: gOR 1.24, CI 1.03–1.50; *p* = 0.03; *I*^2^ = 48%) and higher functional independence (mRS 0–2: RR 1.16, CI 1.02–1.31; *p* = 0.03; *I*^2^ = 0%) compared to EVT alone (Figure 2). Mortality and sICH rates were similar between groups (Supplementary Figure S3). Between 4.5 and 24 h, no difference in disability reduction (gOR 0.95, CI 0.70–1.30; *p* = 0.76; *I*^2^ = 78%) but lower functional independence (RR 0.60, CI 0.40–0.90; *p* = 0.01) was seen with Tenecteplase plus EVT (Figure 2).

**Figure 3:**
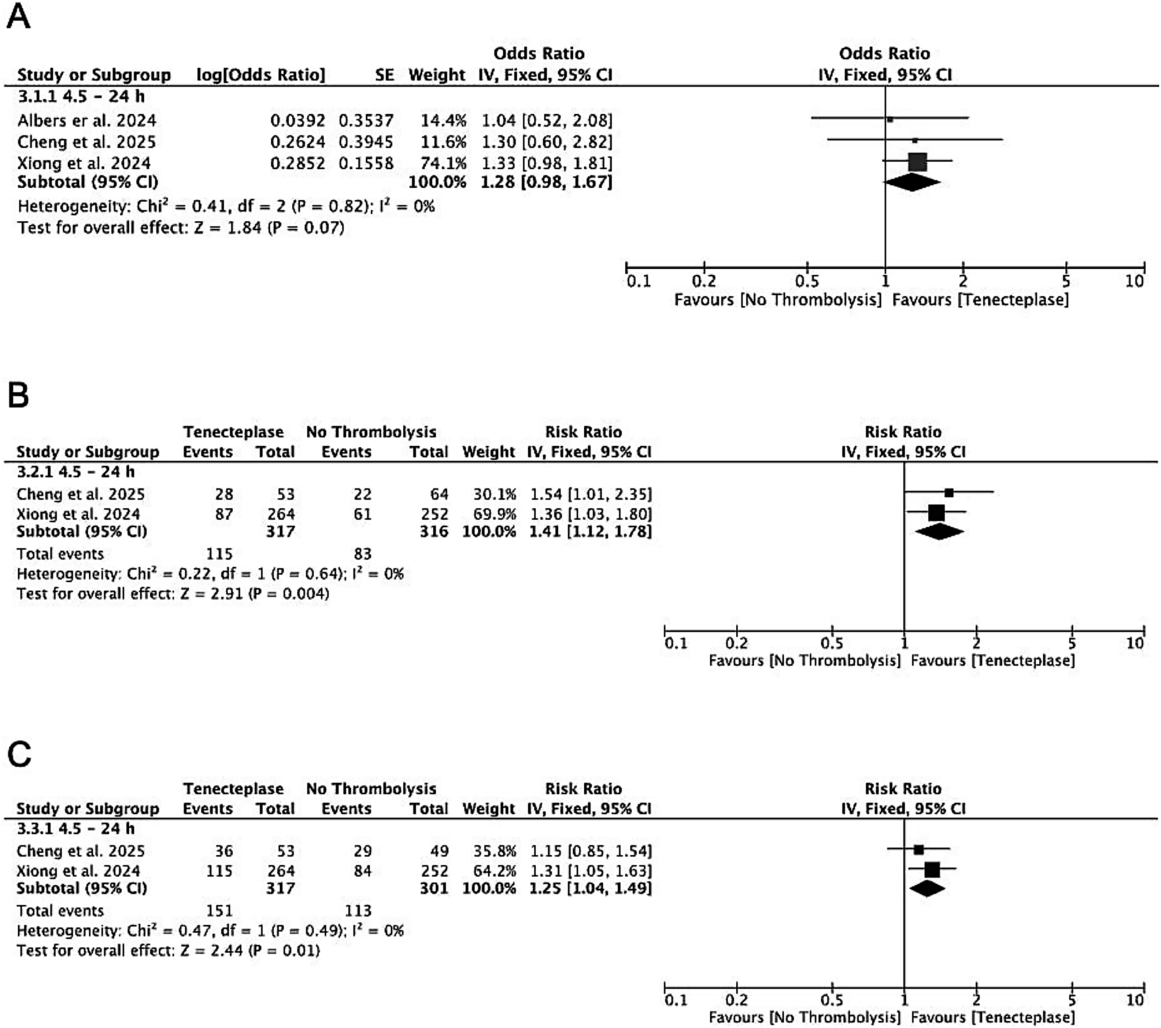
Forest plots of subgroup analyses by treatment timing among patients with LVO not undergoing EVT, showing mRS shift analysis **(A)**, excellent functional outcome (mRS 0–1) **(B)**, and functional independence (mRS 0–2) **(C)**.

In patients who did not undergo EVT and presented after 4.5 h, Tenecteplase was associated with significantly higher rates of excellent outcome (mRS 0–1: RR 1.41, CI 1.12–1.78; *p* = 0.004; *I*^2^ = 0%) and functional independence (mRS 0–2: RR 1.25, CI 1.04–1.49; *p* = 0.01; *I*^2^ = 0%). While there was no significant difference in reducing overall disability compared to no thrombolysis, the effect estimate showed a trend favoring Tenecteplase (gOR 1.28, CI 0.98–1.67; *p* = 0.07; *I*^2^ = 0%) (Figure 3).

## 4 Discussion

Based on currently available aggregate clinical trial data, intravenous Tenecteplase in patients with LVO was associated with improved functional outcomes without increased sICH or mortality compared with those who did not receive IV Tenecteplase. No significant interaction was observed between the benefits of Tenecteplase and whether EVT was carried out or not. In subgroup analyses evaluating the timing of outcomes after Tenecteplase, bridging IVT administered within 4.5 h before EVT was associated with improved functional outcomes and may offer extended-window benefit after 4.5 h for patients with LVO and salvageable brain tissue who lack access to EVT.

Our findings indicate that bridging with Tenecteplase within 4.5 h before EVT is associated with improved outcomes compared with EVT alone, in contrast to prior observations with alteplase reported in the IRIS meta-analysis (7). Within the 4.5-h window, trials such as EXTEND-IA TNK I (9) and BRIDGE-TNK (11) demonstrated that Tenecteplase improved functional outcomes without additional safety concerns, compared with alteplase in EXTEND-IA TNK I and with no thrombolysis in BRIDGE-TNK. Collectively, these data support the expanding clinical use of Tenecteplase across diverse practice settings. Notably, these findings suggest that Tenecteplase in LVO patients before EVT may confer a broader therapeutic window than the approximately 140-min window observed with bridging alteplase. In BRIDGE-TNK, most patients received Tenecteplase beyond 140 min, and the associated benefit remained consistent among those treated after this time point (adjusted RR, 1.33; 95% CI, 1.08–1.64) (11). Although higher early recanalization rates with Tenecteplase compared with alteplase were observed in EXTEND-IA TNK, this finding has not been consistently replicated in subsequent studies (9). In a secondary analysis of the AcT trial, functional and reperfusion outcomes were similar between the 2 agents among patients with LVO (10). Taken together, these findings suggest that the benefits of IVT in LVO are likely multifactorial, reflecting interactions among system-level, patient-level, and agent-related factors, with time to treatment playing a particularly important role rather than intrinsic differences in fibrinolytic activity between agents. Of note, multiple studies have demonstrated that Tenecteplase is associated with shorter door-to-needle times compared with alteplase (23–25). These time savings are likely attributable to practical workflow advantages of Tenecteplase, such as its single-bolus mode of administration. Ongoing trials, such as RESILIENT DIRECT-TNK (NCT05199194), may further validate and broaden these findings.

Beyond 4.5 h, three trials (TIMELESS, CHABLIS-T II, and TRACE III) provided insights into the role of Tenecteplase in LVO patients with salvageable brain tissue based on computed tomography perfusion (CTP) imaging compared to those not treated by Tenecteplase. In the TIMELESS trial, the primary outcome did not differ between groups, though functional independence rates were numerically higher with Tenecteplase (22). In the CHABLIS-T II trial, Tenecteplase improved reperfusion rates in patients with LVO or medium-vessel occlusion without increasing hemorrhage risk or significantly affecting functional outcomes (21). In the TIMELESS and CHABLIS-T II trials, 77 and 54% of patients, respectively, underwent EVT after receiving Tenecteplase. By contrast, the TRACE III trial enrolled patients with LVO who did not have access to EVT; these patients were randomized to Tenecteplase or no thrombolysis, and rates of the primary outcome of excellent recovery were significantly higher with Tenecteplase (12). Rates of sICH after Tenecteplase ranged from 3.3–5.4% in these three trials.

In contrast, the recently published ETERNAL-LVO (Efficacy of Tenecteplase in Large Vessel Occlusion Stroke Within 24 Hours of Symptom Onset) trial (13) did not demonstrate a benefit of Tenecteplase in patients with large-vessel occlusion treated within 24 h of symptom onset. Several factors may explain this discrepancy between ETERNAL-LVO with our findings and with prior literature. The trial faced supply challenges and was terminated early after sufficient evidence had been established supporting Tenecteplase for ischemic stroke management within 4.5 h although the study aimed to evaluate Tenecteplase up to 24 h. In addition, the published report did not provide analyses stratified by EVT status at 4.5 h vs. after this time window. Furthermore, 82% of patients in the control group received alteplase, which may have influenced outcomes. For these reasons, the ETERNAL-LVO data were not included in the present analysis, as the published findings offer limited applicability to the study objective. *Post hoc* and subgroup analyses, particularly evaluating Tenecteplase use in LVO within both 4.5 h and 24 h, are needed to clarify its clinical utility, especially in resource-limited settings.

Globally, IVT and EVT remain underused, with treatment rates of 10 to 15% in high-income countries and less than 2% elsewhere (26). Access to EVT is particularly limited, reaching only 2.8% of patients worldwide, primarily because of shortages in imaging resources and neurointerventional expertise (27, 28). In addition, EVT is frequently unavailable in low- and middle-income countries, where treatment delays often extend beyond the therapeutic window for IVT, leading many patients to miss the opportunity for IVT (29). Against this backdrop, the present findings suggest that Tenecteplase may provide benefit beyond 4.5 h in patients with LVO and salvageable brain tissue demonstrated by computed tomography perfusion, particularly when EVT is unavailable or not indicated. These findings are consistent with the TRACE III trial (12) and the HOPE (Treatment With Intravenous Alteplase in Ischemic Stroke Patients With Onset Time Between 4.5 and 24 Hours) trial (30), both of which demonstrated improved outcomes with tenecteplase or alteplase beyond 4.5 h in patients with penumbral tissue identified on advanced imaging. Unlike TRACE III, where LVO was a strict inclusion criterion, HOPE included a broader population, though a substantial subset (236 of 372 [63.4%]) had LVO (30). Rates of sICH were similar to those in other extended-window trials (3.8% in HOPE and 3.0% in TRACE III). Although detailed vessel-specific analyses are limited by data availability, both trials suggested a greater treatment effect in more distal occlusions of the M1 and M2 segments of the middle cerebral artery compared with internal carotid artery occlusions. Nonetheless, it is important to recognize that advanced imaging is not widely available in many regions with limited access to EVT, particularly in low- and middle-income countries. Although preliminary, these observations highlight opportunities to expand access to reperfusion therapy through optimized care pathways, improved availability of advanced imaging, and the use of telemedicine, while underscoring the need for targeted investments in resources and infrastructure to improve outcomes in underserved populations.

This study has several limitations. First, we conducted a study-level rather than an individual patient–level meta-analysis, which limits the ability to explore detailed interactions between patient characteristics and treatment effects. Second, the potential for selection bias remains, particularly given the strict inclusion criteria applied in the included trials and analyses. Third, substantial heterogeneity was observed across several analyses, which may reflect differences in baseline patient characteristics, eligibility criteria, imaging protocols, and system-level factors among the included studies. To explore potential sources of heterogeneity, we performed prespecified subgroup analyses based on 2 clinically relevant factors: time to treatment and use of endovascular thrombectomy. Nevertheless, further studies with more granular, patient-level data are needed to better delineate these interactions. In addition, the findings should be interpreted with caution in specific clinical contexts, including posterior circulation occlusion and settings with limited access to advanced imaging, such as low- and middle-income countries or resource-constrained health systems. Accordingly, the generalizability of these results may be limited. However, the present findings provide a framework for more targeted future investigations and may inform efforts to advance stroke care across diverse health care settings. Additionally, a trial emulation analysis was also included, which indirectly applied the inclusion and exclusion criteria of a hypothetical randomized trial and used data from previously conducted randomized controlled and high-quality trials. Moreover, the inclusion criteria specified studies in which only a small proportion of patients in the comparator group received another thrombolytic agent. Although this could introduce potential confounding, prespecified sensitivity analyses excluding THE CHABLIS-T II trial were performed and yielded consistent results. Detailed analyses of symptomatic and asymptomatic intracerebral hemorrhage across subgroups were not available.

## 5 Conclusion

In this meta-analysis of RCT data, intravenous Tenecteplase was associated with improved functional outcomes without an increased risk of sICH or mortality among patients with LVO. Treatment effects were consistent regardless of EVT status. In timing-based subgroup analyses, bridging IVT with Tenecteplase administered within 4.5 h before EVT was associated with improved functional outcomes. In addition, these findings suggest a potential benefit of intravenous Tenecteplase beyond 4.5 h in selected patients with LVO and salvageable brain tissue based on advanced brain imaging who do not have access to EVT.

## Data Availability

The original contributions presented in the study are included in the article/Supplementary material, further inquiries can be directed to the corresponding authors.
