# Supplementary material for: Tenecteplase in large-vessel occlusion with or without thrombectomy: a meta-analysis
Source: Front Neurol. 2026 Jan 13;16:1715049. doi: 10.3389/fneur.2025.1715049 (PMC12834819; doi:10.3389/fneur.2025.1715049)

Supplementary Material

This supplementary material has been provided by the authors to give readers additional information about their work.

**eAppendix 1.** Algorithm Used in MEDLINE Search

(("tenecteplase"[MeSH Terms] OR "tenecteplase"[All Fields]) AND (("thrombectomy"[MeSH Terms] OR "thrombectomy"[All Fields] OR "thrombectomies"[All Fields]) OR ("endovascular"[All Fields] AND ("therapy"[All Fields] OR "procedure"[All Fields] OR "treatment"[All Fields])) OR ("mechanical"[All Fields] AND "thrombectomy"[All Fields]))) AND (("large vessel occlusion"[All Fields] OR "LVO"[All Fields] OR ("large"[All Fields] AND "vessel"[All Fields] AND "occlusion"[All Fields])) OR ("stroke"[MeSH Terms] OR "stroke"[All Fields] OR "ischemic stroke"[All Fields]))

Results = 271

**eAppendix 2.** Algorithm Used in Embase Search

('tenecteplase'/exp OR tenecteplase:ti,ab) AND (('thrombectomy'/exp OR thrombectomy:ti,ab OR thrombectomies:ti,ab) OR ('endovascular procedure'/exp OR endovascular:ti,ab OR 'mechanical thrombectomy':ti,ab)) AND (('large vessel occlusion':ti,ab OR lvo:ti,ab) OR ('stroke'/exp OR stroke:ti,ab OR 'ischemic stroke':ti,ab)) AND ([clinical trial]/lim OR [randomized controlled trial]/lim)

Results = 222

**eAppendix 3.** Algorithm Used in Web of Science Search

(ALL=(stroke) OR ALL=(ischemic stroke)) AND (ALL=(large vessel occlusion) OR ALL=(LVO)) AND

(ALL=(Tenecteplase) OR ALL=(TNK)) AND (ALL=(endovascular) OR ALL=(thrombectomy) OR ALL=(EVT))

Results = 165

**eAppendix 4.** Algorithm Used in Cochrane Library Search

(“Stroke” OR “LVO” OR “Large vessel occlusion”) AND (“Tenecteplase” OR “TNK”) AND (“Thrombectomy” OR “EVT” OR “Endovascular”)

Results = 37

**Table S1.** Risk-of-Bias assessment using the RoB2 tool.

| **Study ID** | **Randomization** | **Deviation from intended intervention** | **Missing outcome** | **Measurement of the outcome** | **Selection bias** | **Overall** |
| --- | --- | --- | --- | --- | --- | --- |
| Qiu et al. 2025  (BRIDGE-TNK) | Low risk | Some concerns | Low risk | Low risk | Low risk | Some concerns |
| Cheng et al. 2025 (CHABLIS-T II) | Low risk | Some concerns | Low risk | Low risk | Low risk | Some concerns |
| Xiong et al. 2024 (TRACE-III) | Low risk | Low risk | Low risk | Low risk | Low risk | Low risk |
| Albers et al. 2024 (TIMELESS) | Low risk | Low risk | Low risk | Low risk | Low risk | Low risk |

**Table S2.** Risk-of-Bias assessment using the ROBINS-I tool.

| **Study ID** | **Preintervention** | | **At intervention** | **Postintervention** | | | | **Overall** |
| --- | --- | --- | --- | --- | --- | --- | --- | --- |
|  | **Confounding** | **Selection of participants into the study** | **Classification of intervention** | **Deviation from intended intervention** | **Missing data** | **Measurement of outcome** | **Selection of reported results** |  |
| Altersberger et al. 2025 | Low | Low | Moderate ^a^ | Low | Low | Low | Low | Moderate |

^a^ The selection variable (IV thrombolysis) was probably associated with the outcome

**Figure S1.** Flow chart on the selection of eligible studies

**Figure S2.** Forest plots of safety outcome in patients with large-vessel occlusion treated with vs without intravenous Tenecteplase.

1. **Symptomatic intracranial hemorrhage**


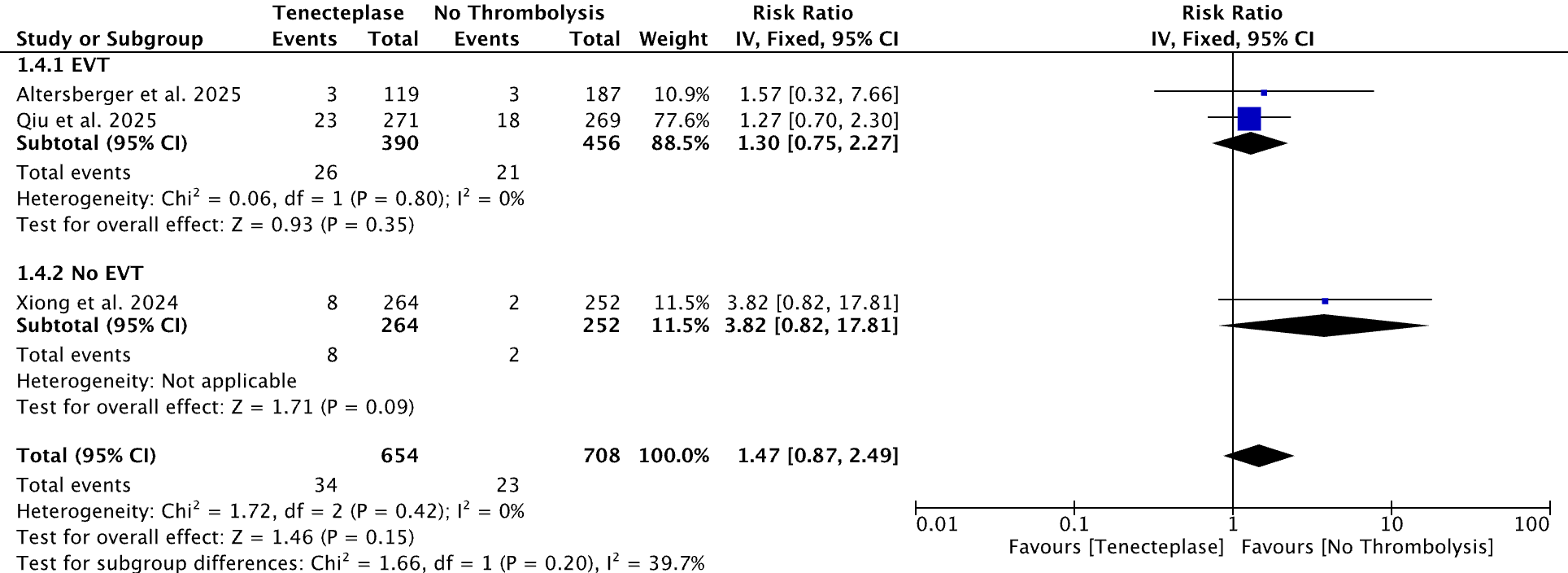


1. **Mortality**


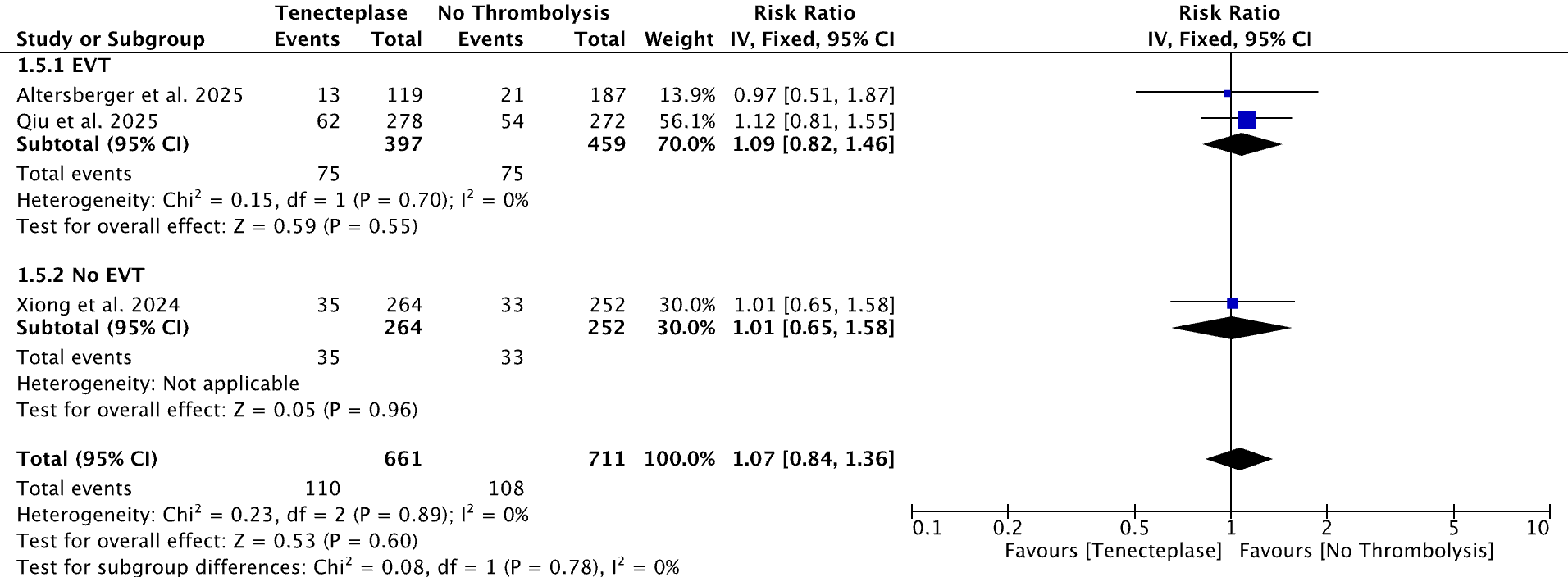


**Figure S3.** Forest plots of the sensitivity analysis after excluding the CHABLIS-T II trial.

1. **Shift analysis in the mRS score**


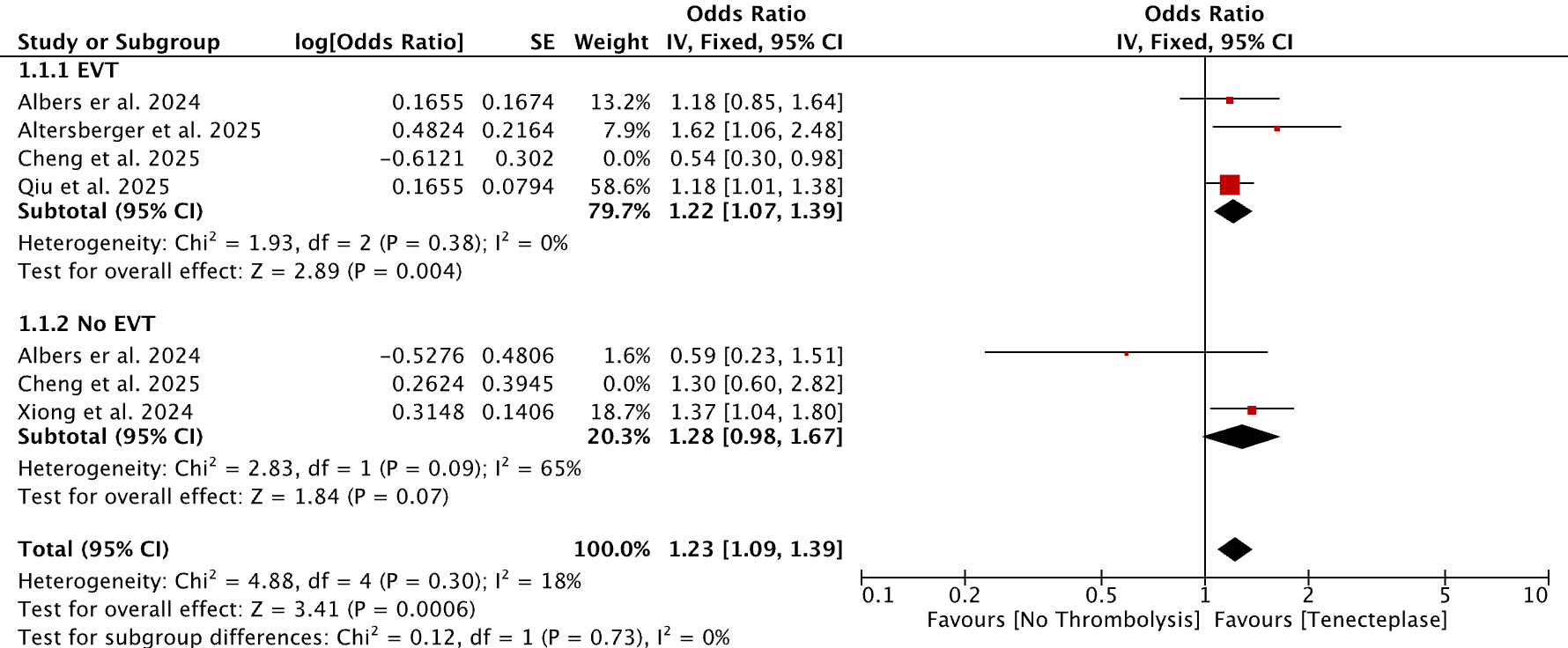


1. **Excellent functional outcome (mRS 0-1)**


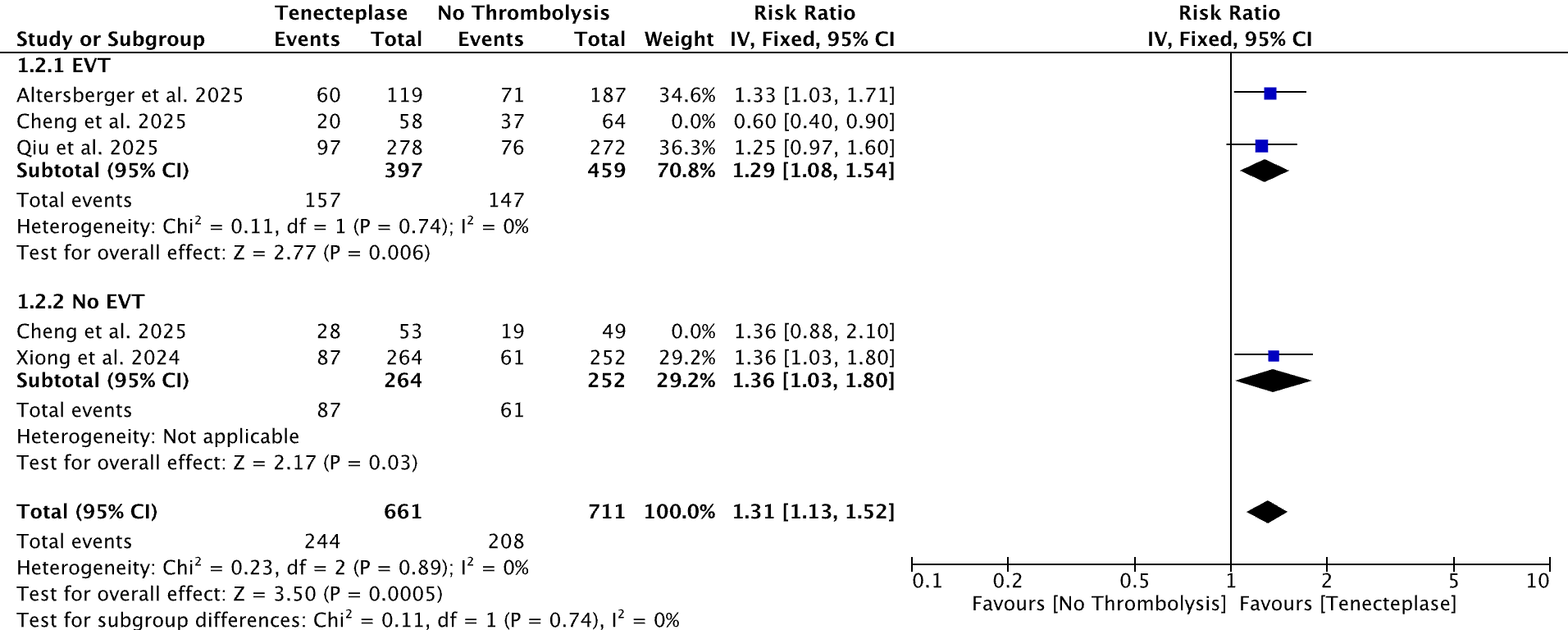


1. **Functional independence (mRS 0-2)**


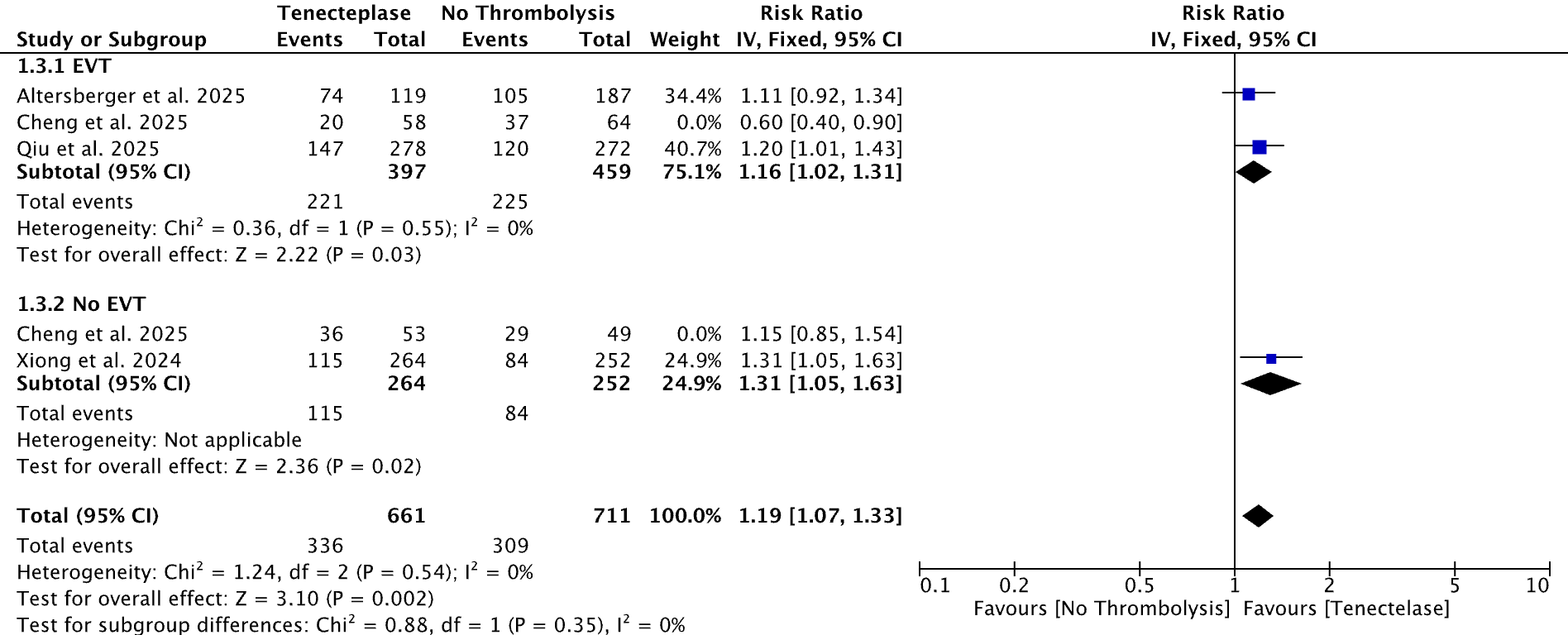


**Figure S4.** Forest plots of the subgroup analysis for safety outcome in patients with large-vessel occlusion treated with Tenecteplase plus endovascular thrombectomy versus endovascular thrombectomy alone treated within 4.5 hours.

1. **Symptomatic intracranial hemorrhage**


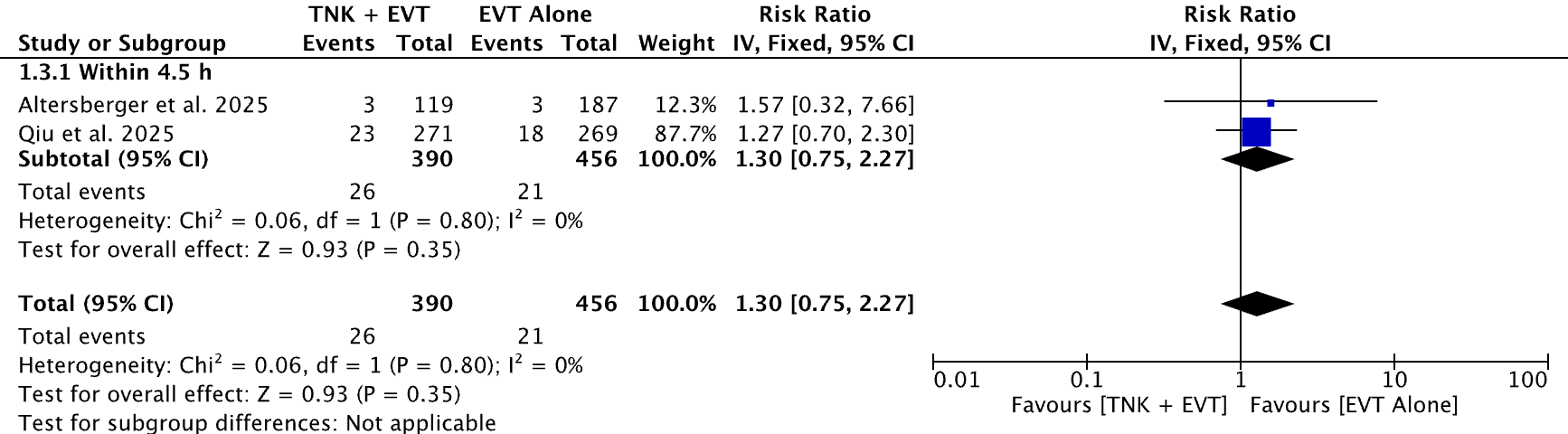


1. **Mortality**


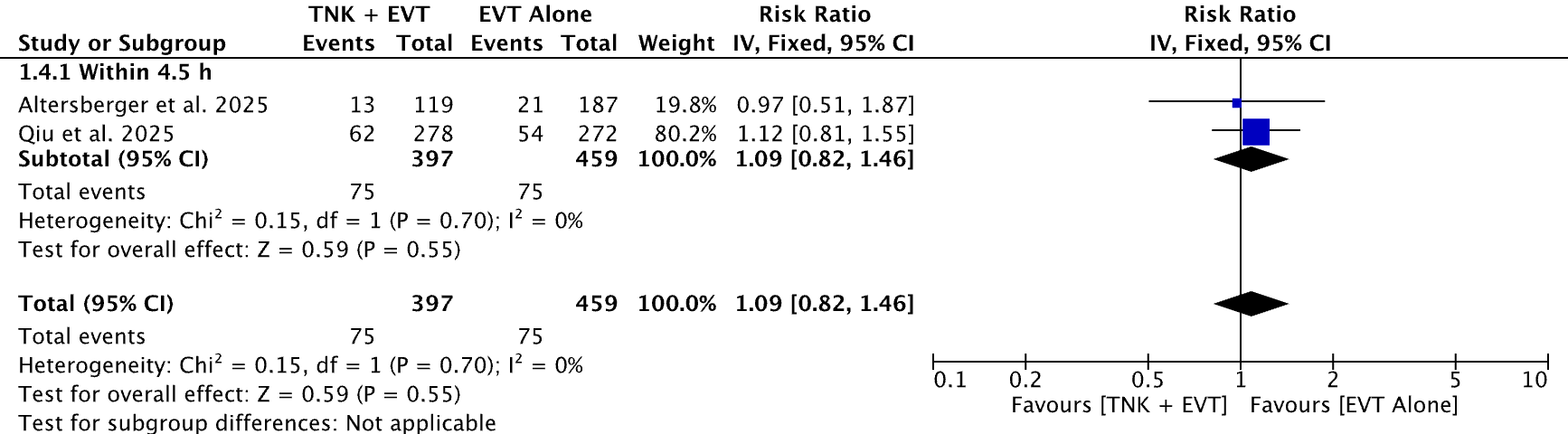

Supplement: Supplementary file 1 [file Data_Sheet_1.DOCX]
